# Quantitative LC-MS/MS Analysis of Proteins Involved in Metastasis of Breast Cancer

**DOI:** 10.1371/journal.pone.0130760

**Published:** 2015-07-15

**Authors:** Rieko Goto, Yasushi Nakamura, Tomonori Takami, Tokio Sanke, Zenzaburo Tozuka

**Affiliations:** 1 Department of Clinical Laboratory Medicine, Wakayama Medical University,Wakayama, Japan; 2 JCL Bioassay Corporation, Nishiwaki, Hyogo, Japan; 3 Graduate School of Pharmaceutical Science Osaka University, Suita, Osaka, Japan; University of North Carolina School of Medicine, UNITED STATES

## Abstract

The purpose of this study was to develop quantitative liquid chromatography-tandem mass spectrometry (LC-MS/MS) methods for the analysis of proteins involved in metastasis of breast cancer for diagnosis and determining disease prognosis, as well as to further our understand of metastatic mechanisms. We have previously demonstrated that the protein type XIV collagen may be specifically expressed in metastatic tissues by two dimensional LC-MS/MS. In this study, we developed quantitative LC-MS/MS methods for type XIV collagen. Type XIV collagen was quantified by analyzing 2 peptides generated by digesting type XIV collagen using stable isotope-labeled peptides. The individual concentrations were equivalent between 2 different peptides of type XIV collagen by evaluation of imprecise transitions and using the best transition for the peptide concentration. The results indicated that type XIV collagen is highly expressed in metastatic tissues of patients with massive lymph node involvement compared to non-metastatic tissues. These findings were validated by quantitative real-time RT-PCR. Further studies on type XIV collagen are desired to verify its role as a prognostic factor and diagnosis marker for metastasis.

## Introduction

The 5-year breast cancer survival rate is generally greater than 90% but it is roughly 55% in women with numerous lymph node metastases [1–3]. The prognosis of breast cancer associated with nodal metastasis, age, tumor size (pT), histological grade of tumor, estrogen and progesterone receptors status, human epidermal growth factor receptors (HERs) status, and breast cancer (BRCA) gene mutations. Nodal status (including number and location of nodes) correlates with disease-free and overall survival better than any other prognostic factor. Also, long-term prognosis depends on tumor stage. Treatment of breast cancer relies on surgical excision and various forms of systemic therapy. However, as metastasized and recurrent breast cancers (especially breast cancer with massive nodal metastasis) are difficult to cure, the development of new therapeutic agents including molecular targeted drugs is awaited. We have searched for the role of lymph node- and metastasis-related biomarkers [4]. Lymph nodes are the first site of metastasis for several carcinomas, and the extent of lymph node metastasis is a major criterion for evaluating patient prognosis.

In previous studies in which we screened for metastasis-specific proteins, breast cancer tissues from a non-metastasis group and a massive lymph node metastasis group were compared based on comprehensive analysis using 2-dimensional liquid chromatography-tandem mass spectrometry (2D LC-MS/MS); 34 proteins were screened as specific candidate proteins involved in metastasis by 2D LC-MS/MS analysis. Type XIV collagen in particular was found to be selectively expressed in specimens from the massive lymph node metastasis group; therefore the protein may be a potential biomarker of metastasis and useful for evaluating patient prognosis. The difference in expression was confirmed by immunohistochemistry [5–8]. However, in the 2D LC-MS/MS analysis, the 34 proteins were screened by protein score based on XCorr, which is measured from mass and MS/MS peaks and correlates with peptide sequence [9, 10]; the proteins have not yet been demonstrated by quantitative analysis. The discovery of candidate biomarker proteins is often hampered by false-positive results caused by imprecision, and quantification of targeted proteins is needed for screening of candidate protein biomarkers [9–15]. Therefore, development of quantitative of methods for directly measuring expression of these proteins, including type XIV collagen, could be useful for diagnosis, prognosis, and understanding of the mechanisms of metastasis. Multiple reaction monitoring (MRM)-based and selected reaction monitoring (SRM)-based quantification using LC-MS/MS have been used for quantitative simultaneous analysis of multiple proteins without antibodies [16–20]. In MRM and SRM analysis, ion suppression of small molecules–i.e., reduction of sensitivity due to ionization of endogenous substances in the biological fluid matrix–is a major issue that must be considered. To avoid the influence of ion suppression on quantification, stable isotope-labeled peptide, which co-elutes with the endogenous peptide, is used [21–23]. The evaluation of imprecise transitions is important in protein quantification because interference in biological fluids due to impurities affects quantification [24, 25].

Our purpose in the present study was to develop quantitative LC-MS/MS methods for the analysis of proteins involved in metastasis of breast cancer, so as to facilitate diagnosis and determination of disease prognosis, as well as to shed light on the mechanisms involved in metastasis. In this preliminary study, specific candidate proteins were selected, and as model of quantification of the proteins, type XIV collagen was quantified with stable isotope-labeled peptides by LC-MS/MS and the expression compared between massive lymph node metastasis tissues and non-metastasis tissues.

## Materials and Methods

### 1. Ethics statement

Institutional review board of Wakayama Medical University approved this study, and all patients had given written informed consent.

### 2. Subjects and samples

Fresh tumor tissue specimens of primary lesions were obtained after surgical resection from 220 patients with primary breast cancer. Each tumor specimen was stored at −80°C prior to protein purification. To detect the molecules responsible for lymph node metastasis, we compared patients with massive lymph node metastasis and patients with absolutely no lymph node metastasis. From 220 consecutive patients who underwent surgical resection of their breast cancers, we selected 13 patients with more than 10 lymph node metastases (pN3) and 6 patients with no lymph node involvement at all (pN0(mol-)). Patients were carefully classified into 2 groups according to the degree of lymph node metastasis. The non-metastasis group had invasive cancer but was free of lymph node metastasis; no lymph node metastases were detected on pathological examination, and the samples were confirmed to be negative for cytokeratin 19 by the one-step nucleic acid amplification (OSNA) method. The other group consisted of patients with massive lymph node involvement with more than 10 lymph node metastases detected on pathological examination. This type of cancer is highly aggressive and generally has an extremely low survival rate. In this study, the non-metastasis group was comprised of six cases (subjects 1–6), and the massive lymph node metastasis group included 13 cases (subjects 7–19).

### 3. Quantitative LC-MS/MS analysis

#### 3.1. Protein purification

Each tissue specimen (a slice ≈600 μm thick) was dissociated into single cells by addition of 150 μL collagenase solution [500 μg/mL collagenase, 137 mM NaCl, 10 mM HEPES (pH 7.5) 5 mM KCl, 5 mM CaCl_2_, 4 mM NaHCO_3_, 0.8 mM Na_2_HPO_4_.2H_2_O, 0.5 mM NaH_2_PO_4_.H_2_O, and 2 μL protease inhibitor cocktail], followed by incubation at 37°C for 10 min. The cells were washed with 450 μL minimum essential medium (MEM) and separated by centrifugation (10 × g, for 2 min at 4°C). The supernatant was removed, and the residue containing the cells was washed twice in 600 μL PBS buffer. The cells were recovered by centrifugation (160 × g, for 5 min at 4°C), resuspended in 600 μL buffer A [10 mM HEPES-NaOH (pH 7.9), 10 mM KCl, 1.5 mM MgCl_2_, 0.5 mM DTT, 0.5 mM PMSF, 2 μL protease inhibitor cocktail], and recovered again by centrifugation (1000 × g, for 10 min at 4°C). The residue containing the cells was placed in 340 μL of new buffer A and homogenized slowly by rotating 20 times on ice at 70 rpm. The homogenates were centrifuged at 1000 × g for 10 min at 4°C. The supernatant containing the cytoplasm was stored at −80°C until analysis. The total protein content in purified samples was determined by a protein assay based on the Bradford method (Bio-Rad Laboratories, Hercules, CA, USA), using bovine serum albumin as a standard.

#### 3.2. Protein digestion

Purified samples were diluted with 50 mM NH_4_HCO_3_ to 40 μL total volume and reduced with 10 μL of 100 mM DTT in 50 mM NH_4_HCO_3_ for 60 min at 50°C. Cysteine residues were alkylated with 20 μL of 100 mM iodoacetamide in 50 mM NH_4_HCO_3_ in the dark at room temperature for 30 min. Each mixture was proteolyzed to peptides with 20 μL of 5 μg/mL trypsin (Promega, Madison, WI, USA) in 50 mM NH_4_HCO_3_ for 16 h at 37°C. Proteolysis was stopped by addition of 10 μL of 10% formic acid.

#### 3.3. Protein and peptide selection for quantification

Proteins with higher proteotypic peptide response were screened as specific candidate proteins by in silico digestion using Pinpoint software (version 1.0 Thermo Fisher ScientiFIc). MS/MS spectra of 4 to 27 amino acid peptides were composited with in silico digestion. MS/MS spectra of 2D LC-MS/MS were compared to the composite MS/MS spectra with Xcorr values above 2.0, 2.0, and 3.3 for 1+, 2+, and 3+ charge states of the peptide. Five MS/MS spectra with high intensity in each peptide of the candidate protein were selected from m/z 600–1250 for protein quantification.

#### 3.4. Peptide selection by MRM analysis without internal standard

To confirm differential abundance of the specific candidate proteins, purified samples (7 μg total protein) from each subject were digested, and the digestion solution (0.4 μg total protein) was eluted using a linear gradient of 5–65% acetonitrile containing 0.1% formic acid at a flow rate of 0.2 mL/min for 15 minutes on an Ultra-Fast Liquid Chromatograph (Shimadzu, Kyoto, Japan) that was equipped with a C18 column (Jupiter C18, 5 μm, 300 Å, 2.0 × 150 mm; Phenomenex, Torrance, CA, USA). The peptides of each subject’s sample were analyzed repeatedly in MRM mode using QTRAP 5500 (AB SCIEX, Foster City, CA, USA). Only peptide peaks shown at the same retention time in a minimum of 3 MRM chromatograms of the same peptide were deemed as target peptide peaks originating from the protein [20]. Based on the difference in peptide abundance between the 2 groups, candidate proteins whose retention times were the same among multi-transition chromatograms and transitions with peak height greater than 1000 cps were selected. Three transitions for each target peptide of the candidate proteins were selected based on the formation of higher intensity product ions.

#### 3.5. Quantification with stable isotope-labeled peptides

For the development of quantitative analytical methods, 2 stable isotope-labeled peptides AQUA peptides corresponding to type XIV collagen (purchased from Thermo Fisher Scientific) were used as internal standards.

Purified samples in triplicate (6 **μ**g total protein per replicate) from each subject were digested in the presence of stable isotope-labeled peptides (2 pmol), and the digestion samples (3 **μ**g total protein) were quantitatively analyzed by MRM using stable isotope-labeled peptides. Equipment used was the same as that for MRM analysis without internal standard. LC methods, masses, collision energy (CE), declustering potential (DP), and collision cell exit potential (CXP) were optimized for quantitative analysis using each AQUA peptide. Elution was performed at a flow rate of 0.2 mL/min with a linear gradient of 3% to 65% acetonitrile containing 0.1% formic acid for 29 minutes. Initially, we set the following 2 criteria to determine the concentration of a protein: 1) the retention times of peaks in multichromatograms of transitions should be the same between endogenous peptide, thereby confirming that the peptide originated from the target protein [20]; and 2) the transition should have an average peak height for endogenous peptide above 1000 cps in the massive lymph node metastasis group.

In addition to these criteria, we subsequently added a third to reduce the influence of matrix-derived interference, which became apparent later. The third criterion was exclusion of a transition from the peptide concentration calculation if the evaluation of imprecise transitions was influenced by interference. For example, when there is a transition for a subject whose coefficient of variation (CV) of intra-assay reproducibility (n = 3) is above 50, the transition should be excluded from peptide concentration calculations. Finally, the best transition with high specificity and high sensitivity was selected from transitions which met the 3 criteria, and concentrations of the best transition were used as the peptide concentrations. The peptide concentrations were interpreted as protein concentrations.

### 4. Validation by Quantitative real time Reverse Transcription Polymerase Chain Reaction (real-time RT-PCR)

Messenger RNA (mRNA) was extracted from fresh frozen tumor tissue samples using QuickPrep micro mRNA purification kit (Amersham Biosciences, Buckinghamshire, UK) according to the protocol provided by the manufacturer. mRNA was reverse-transcribed to single-strand cDNA using Oligo-(dT)20 primers and Thermoscript (Invitrogen, Tokyo, Japan). The RT reaction was performed at 55°C for 60 min, followed by heating at 85°C for 5 min. We confirmed that only carcinoma tissue was included in the freshly frozen thyroid tissue samples by viewing cryostat sections. Transcriptional levels of collagen XIV (COL14A1) were measured by quantitative real-time PCR using universal TaqMan PCR reagents, and the reactions were recorded and analyzed using an ABI Prism 7000 sequence detector equipped with a 96-well thermal cycler (Applied Biosystems, CA, USA). The gene transcript level of each sample (ΔCt) was normalized to its GAPDH transcript content, which was used as an internal control. All experiments were performed in triplicate and the mean values were calculated (meanΔCt). Then, inverse (ΔΔCt) values were calculated using Mann-Whitney U test for statistical testing. The cDNA templates were subjected to a 5-min initial denaturation at 95°C prior to 40 cycles of PCR (95°C for 15 sec and 60°C for 1 min, per cycle). The primer and probe mixture for COL14A1 or GAPDH was purchased from Applied Biosystems, and PCR was carried out according to the manufacturer’s protocol.

## Results

### 1. Quantitative LC-MS/MS analysis

#### 1.1 Protein and peptide selection for quantification

By comprehensive analysis using 2D LC-MS/MS, 34 proteins were screened as specific candidate proteins expressed highly in massive lymph node metastasis breast cancer [5]. From these 34 proteins, those whose transitions were confirmed by Pinpoint software were selected for MRM analysis; i.e., 104 MRM transitions for 22 peptides of 7 proteins (4–5 transitions per peptide).

#### 1.2. Peptide selection by MRM analysis without internal standard

In order to confirm differential abundance of a specific candidate protein, the 104 MRM transitions for 22 peptides of 7 proteins from each subject were analyzed using a QTRAP5500 system. From the results of the 104 MRM transitions, 58 MRM transitions for 13 peptides of 7 proteins (3–5 transitions per peptide) were selected as candidates, since the fragment ions had higher intensities. From the results of the 58 MRM transitions, 28 MRM transitions for 9 peptides of 7 proteins (3 transitions per peptide) were selected as the best transitions for each peptide, since the fragment ions had higher intensities. In the results of the 28 MRM transitions for 9 peptides of 7 proteins, the retention time of the peaks differed among multiple transition of 1 peptide. Therefore, the protein for which the retention time between peptides differed was excluded, and the remaining 6 proteins were selected as specific candidate proteins. The proteins were type XIV collagen, hexokinase I, MSTP161, angiomotin, alpha-2 type I collagen, and alpha-1 type I collagen.

#### 1.3. Quantification with stable isotope-labeled peptides

Type XIV collagen was measured by quantitative LC-MS/MS with stable isotope-labeled peptides via analyzing 12 MRM transitions for 2 peptides generated by digesting type XIV collagen (3 transitions per peptide) within 1 injection. The quantitative results of purified samples in triplicate from each subject (subjects 1–6, non metastasis; subjects 7–18, massive lymph node metastasis) are listed in Table 1. Seven samples were diluted as necessary, and the sample for subject 19 was not analyzed due to insufficient quantity. All transitions had the same retention time between the endogenous peptide and the stable isotope-labeled peptides, and the average peak heights of endogenous peptide in the massive lymph node metastasis group were greater than 1000 cps, which satisfied the criteria.

**Table 1:** Concentrations of type XIV collagen determined by quantitative LC-MS/MS analysis with stable isotope-labeled peptides.

			**Peptide 1: ITWDPPSSPVK, Stable isotope-labeled peptides of Peptide 1: I (^13^C_6_, ^15^N) TWDPPSSPVK**											
			**Q1/Q3 transition of Peptide 1 (Q1/Q3 transition of AQUA Peptide)**											
			**614/711 (617/711)**			**614/826 (617/826)**			**614/1013 (617/1013)**			**Among transitions**		**Peptide**
**Group**	**Subject Number**	**Dilution Factor**	**Average Retention Time** *^1^ **(min)**	**Average Conc.** *^2^ **(fmol/μg)**	**CV of intra-assay** *^3^ **(%)**	**Average Retention Time** *^1^ **(min)**	**Average Conc.** *^2^ **(fmol/μg)**	**CV of intra-assay** *^3^ **(%)**	**Average Retention Time** *^1^ **(min)**	**Average Conc.** *^2^ **(fmol/μg)**	**CV of intra-assay** *^3^ **(%)**	**Average Conc.** *^6^ **(fmol/μg)**	**Difference (%)**	**Transition 614/711 (617/711)**
**Non-metastasis**	**1**	**1**	**18.6**	**0.9**	**12.5**	**-**	**N.D.**	**N.C.**	**18.7** ^**#1**^	**1.5** ^**#1**^	**173.2** *^5^	**0.5** *^5^	**100.0** *^5^	**0.9**
	**2**	**1**	**18.4**	**2.8**	**7.2**	**18.3** ^**#1**^	**0.8** ^**#1**^	**173.2** *^5^	**18.5**	**6.2**	**31.9**	**1.8** *^5^	**71.9** *^5^	**2.8**
	**3**	**1**	**18.4**	**1.5**	**10.7**	**-**	**N.D.**	**N.C.**	**18.5**	**7.1**	**6.6**	**0.7** *^5^	**100.0** *^5^	**1.5**
	**4**	**1**	**-**	**N.D.**	**N.C.**	**-**	**N.D.**	**N.C.**	**18.4** ^**#2**^	**21.2** ^**#2**^ ^,^ *^4^	**96.7** *^4^ ^,^ *^5^	**N.D.**	**N.C.** *^5^	**N.D.**
	**5**	**1**	**18.5**	**1.8**	**12.6**	**-**	**N.D.**	**N.C.**	**18.6**	**6.2**	**15.3**	**0.9** *^5^	**100.0** *^5^	**1.8**
	**6**	**2**	**18.5**	**6.7**	**11.6**	**18.5**	**7.1**	**18.8**	**18.5**	**8.8**	**30.1**	**6.9**	**-6.2**	**6.7**
**Massive lymph node metastasis**	**7**	**1**	**-**	**N.D.**	**N.C.**	**-**	**N.D.**	**N.C.**	**18.6**	**277.8** *^4^	**50.3** *^4^	**N.D.**	**N.C.** *^5^	**N.D.**
	**8**	**1**	**18.3** ^**#2**^	**0.5** ^**#2**^	**86.7** *^5^	**-**	**N.D.**	**N.C.**	**18.5**	**5.9**	**10.2**	**0.2** *^5^	**100.0** *^5^	**0.5**
	**9**	**1**	**18.5**	**11.9**	**3.2**	**18.5**	**13.2**	**9.4**	**18.5**	**14.2**	**15.8**	**12.5**	**-10.7**	**11.9**
	**10**	**2**	**18.6**	**13.5**	**2.5**	**18.6**	**13.6**	**5.4**	**18.6**	**18.9**	**2.9**	**13.6**	**-0.8**	**13.5**
	**11**	**2**	**18.5**	**26.0**	**6.6**	**18.5**	**27.6**	**5.1**	**18.5**	**32.8**	**15.3**	**26.8**	**-6.2**	**26.0**
	**12**	**1**	**18.4**	**4.1**	**8.2**	**18.4**	**5.0**	**9.2**	**18.5**	**9.9**	**39.5**	**4.5**	**-21.5**	**4.1**
	**13**	**2**	**18.4**	**6.7**	**17.4**	**18.4**	**7.4**	**10.5**	**18.4**	**12.2**	**12.2**	**7.1**	**-10.3**	**6.7**
	**14**	**4**	**18.4**	**16.7**	**4.8**	**18.4**	**18.2**	**6.6**	**18.4**	**19.1**	**6.6**	**17.5**	**-9.0**	**16.7**
	**15**	**1**	**18.4**	**12.0**	**5.1**	**18.4**	**14.1**	**11.3**	**18.4**	**18.5**	**28.9**	**13.1**	**-17.1**	**12.0**
	**16**	**1**	**18.6**	**9.7**	**6.7**	**18.6**	**10.0**	**0.5**	**18.6**	**11.7**	**32.1**	**9.8**	**-3.4**	**9.7**
	**17**	**2**	**18.5**	**13.4**	**7.0**	**18.5**	**13.0**	**4.5**	**18.5**	**16.2**	**11.1**	**13.2**	**2.8**	**13.4**
	**18**	**2**	**18.3**	**10.5**	**12.1**	**18.4**	**11.4**	**27.9**	**18.3**	**13.3**	**6.1**	**10.9**	**-8.9**	**10.5**
			**Peptide 2: ASAHAITGPPTELITSEVTAR, Stable isotope-labeled peptides of Peptide 2: ASAHAI (** ^**13**^ **C**_**6**_, ^**15**^ **N) TGPPTELITSEVTAR**											
			**Q1/Q3 transition of Peptide 2 (Q1/Q3 transition of AQUA Peptide)**											
			**708/624 (710/627)**			**708/763 (710/763)**			**708/877 (710/877)**			**Among transitions**		**Peptide**
**Group**	**Subject Number**	**Dilution Factor**	**Average Retention Time** *^1^ **(min)**	**Average Conc.** *^2^ **(fmol/μg)**	**CV of intra-assay** *^3^ **(%)**	**Average Retention Time** *^1^ **(min)**	**Average Conc.** *^2^ **(fmol/μg)**	**CV of intra-assay** *^3^ **(%)**	**Average Retention Time** *^1^ **(min)**	**Average Conc.** *^2^ **(fmol/μg)**	**CV of intra-assay** *^3^ **(%)**	**Average Conc.** *^6^ **(fmol/μg)**	**CV among transitions (%)**	**Transition 708/763 (710/763)**
**Non-metastasis**	**1**	**1**	**-**	**N.D.**	**N.C.**	**-**	**N.D.**	**N.C.**	**-**	**N.D.**	**N.C.**	**N.D.**	**N.C.**	**N.D.**
	**2**	**1**	**20.0**	**1.9**	**6.0**	**20.0**	**1.9**	**13.2**	**20.1**	**2.2**	**34.6**	**2.0**	**9.4**	**1.9**
	**3**	**1**	**-**	**N.D.**	**N.C.**	**20.0**	**1.5**	**15.8**	**20.0** ^**#1**^	**0.8** ^**#1**^	**173.2** *^5^	**0.7** *^5^	**99.3** *^5^	**1.5**
	**4**	**1**	**19.9** ^**#2**^	**1.1** ^**#2**^	**87.6** *^5^	**19.9**	**1.5**	**27.4**	**20.0** ^**#1**^	**0.6** ^**#1**^	**173.2** *^5^	**1.1** *^5^	**39.8** *^5^	**1.5**
	**5**	**1**	**-**	**N.D.**	**N.C.**	**20.1**	**1.2**	**4.3**	**20.0** ^**#1**^	**0.4** ^**#1**^	**173.2** *^5^	**0.5** *^5^	**112.5** *^5^	**1.2**
	**6**	**2**	**20.0**	**3.8**	**23.6**	**20.1**	**4.8**	**13.3**	**20.0**	**4.6**	**18.3**	**4.4**	**12.5**	**4.8**
**Massive lymph node metastasis**	**7**	**1**	**20.0**	**3.9**	**3.0**	**20.1**	**4.0**	**3.8**	**20.1**	**3.9**	**9.1**	**3.9**	**1.2**	**4.0**
	**8**	**1**	**-**	**N.D.**	**N.C.**	**20.0** ^**#1**^	**0.3** ^**#1**^	**173.2** *^5^	**-**	**N.D.**	**N.C.**	**0.1** *^5^	**173.2** *^5^	**0.3**
	**9**	**1**	**20.1**	**9.2**	**9.9**	**20.1**	**8.4**	**18.1**	**20.1**	**9.3**	**13.6**	**9.0**	**5.5**	**8.4**
	**10**	**2**	**20.2**	**8.2**	**6.1**	**20.1**	**7.7**	**11.0**	**20.1**	**7.8**	**13.9**	**7.9**	**3.5**	**7.7**
	**11**	**2**	**20.0**	**16.7**	**13.3**	**20.0**	**16.4**	**13.9**	**20.0**	**16.5**	**9.1**	**16.5**	**1.1**	**16.4**
	**12**	**1**	**20.0**	**3.0**	**7.2**	**20.0**	**2.5**	**8.5**	**20.0**	**2.8**	**13.3**	**2.8**	**8.6**	**2.5**
	**13**	**2**	**20.0**	**4.4**	**12.0**	**20.0**	**4.5**	**18.3**	**20.0**	**5.6**	**11.9**	**4.8**	**14.4**	**4.5**
	**14**	**4**	**20.1**	**13.1**	**9.4**	**20.1**	**14.6**	**6.9**	**20.0**	**13.8**	**12.0**	**13.8**	**5.3**	**14.6**
	**15**	**1**	**20.1**	**9.0**	**4.9**	**20.1**	**9.0**	**6.6**	**20.1**	**9.2**	**6.2**	**9.1**	**1.7**	**9.0**
	**16**	**1**	**20.2**	**7.1**	**5.4**	**20.2**	**6.4**	**10.8**	**20.2**	**6.5**	**7.3**	**6.7**	**5.3**	**6.4**
	**17**	**2**	**20.1**	**8.5**	**4.0**	**20.1**	**7.7**	**17.6**	**20.1**	**9.1**	**13.9**	**8.4**	**8.5**	**7.7**
	**18**	**2**	**20.0**	**8.3**	**20.8**	**20.0**	**8.0**	**5.5**	**20.0**	**7.9**	**2.7**	**8.1**	**2.8**	**8.0**

*^1^: Average retention time; Average retention time of triplicate samples (^#1^: Retention time of one sample, ^#2^: Average retention time of duplicate samples.-: All samples were below detection limit.).

*^2^: Average Conc.; Average intra-assay concentration (n = 3) (^#1^: Concentration of one sample, ^#2^: Average concentration of duplicate samples, N.D.: Not detected because concentrations of all triplicate samples were not detected, and the concentration was calculated as 0.0.).

*^3^: CV of intra-assay; Coefficient of variation of intra-assay reproducibility (n = 3) (N.C.: Not calculated because concentrations of all tripricate samples were not detected.)

*^4^: Value including interferences

*^5^: Value including detected samples and non-detected samples

*^6^: Average conc.; Average concentration from 2 transitions (614/711and 614/826) for peptide 1 and 3 transitions (708/624, 708/763, 708/877) for peptide 2.

*^7^: Difference of peptide 1; Difference (%) = (Conc. of 614/711-Conc. of 614/826)×100 /Conc. of 614/711, (N.C.: Not calculated because concentration of 614/711 was 0.)

^#1^: Value of 1 sample because other duplicate samples were not detected.

^#2^: Average of duplicate samples because another sample was not detected.

**Fig 1:**
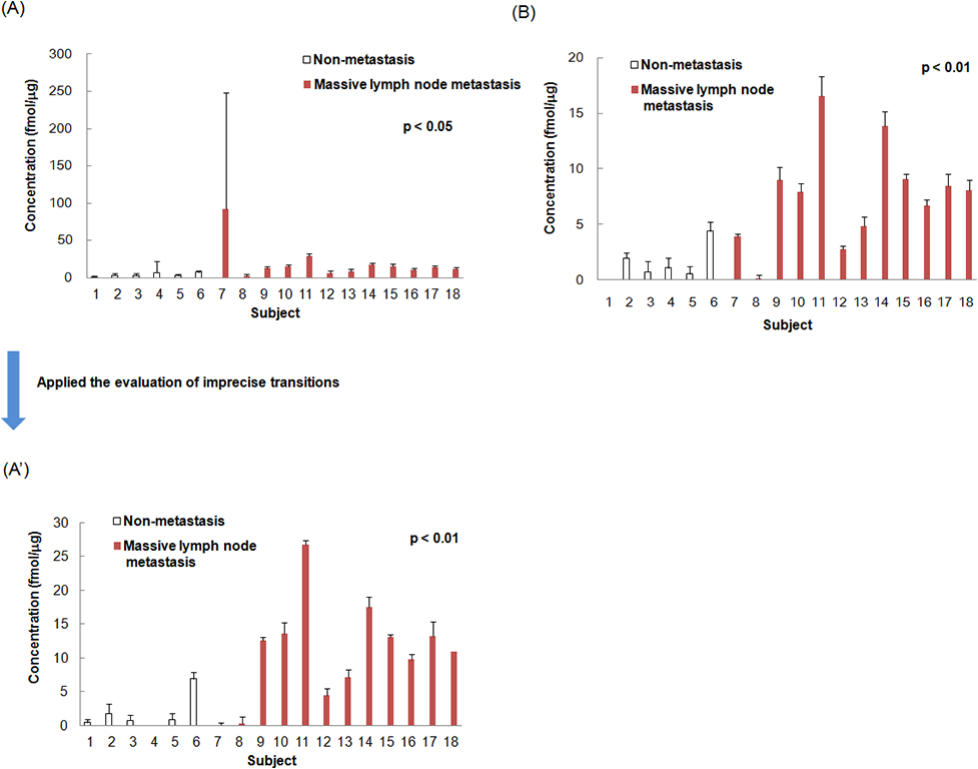
Average intra-assay concentration of peptide ITWDPPSSPVK and peptide ASAHAITGPPTELITSEVTAR of type XIV collagen. Peptides from each subject (subjects 1–6: non metastasis, subjects 7–18: massive lymph node metastasis) were quantified (n = 3) by LC-MS/MS. The average intra-assay concentration and SD of peptide concentrations from individual proteins are calculated from all transitions (614/711, 614/826 and 614/1013) for peptide ITWDPPSSPVK of Protein 12 (A), 2 transitions (614/711 and 614/826) for peptide ITWDPPSSPVK (A'), and all transitions (708/624, 708/763 and 708/877) for peptide ASAHAITGPPTELITSEVTAR (B) of type XIV collagen. By excluding the low specificity transition 614/1013, the concentration of peptide ITWDPPSSPVK was changed (A) to (A'), which correlated with the concentrations of (B). Significant difference between non metastasis and massive lymph node metastasis in (A') and (B).

**Fig 1** shows the average intra-assay concentrations (n = 3) in all transitions (614/711, 614/826 and 614/1013) for peptide ITWDPPSSPVK, 2 transitions (614/711 and 614/826) for peptide ITWDPPSSPVK, and all transitions (708/624, 708/763 and 708/877) for peptide ASAHAITGPPTELITSEVTAR of type XIV collagen. Although, theoretically, the concentration of peptides that originated from the same protein should be the same, the difference in the average intra-assay concentration between peptides ITWDPPSSPVK and ASAHAITGPPTELITSEVTAR of type XIV collagen was remarkable in subjects 4 and 7 (**Fig 1A and 1B**, and **Table 1**). This discrepancy arose from an extremely high concentration of transition 614/1013 of peptide ITWDPPSSPVK.

**Fig 2:**
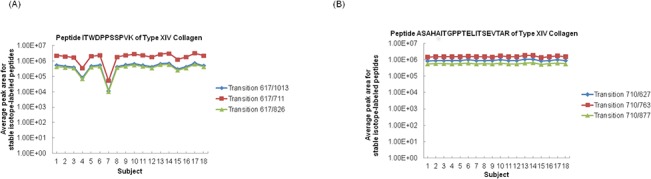
Average peak area of internal standards of peptide ITWDPPSSPVK and peptide ASAHAITGPPTELITSEVTAR. The internal standards of peptide ITWDPPSSPVK and peptide ASAHAITGPPTELITSEVTAR were peptide I (^13^C_6_, ^15^N) TWDPPSSPVK and peptide ASAHAI (^13^C_6_, ^15^N) TGPPTELITSEVTAR, respectively. The peak areas of peptide I(^13^C_6_, ^15^N)TWDPPSSPVK were 8% in subject 4 and 1% in subject 7, which were less than 10% of the values in other subjects (A). The peak area of peptide ASAHAI(^13^C_6_, ^15^N)TGPPTELITSEVTAR was equivalent in all subjects.

**Fig 2A and 2B** show the average of intra-assay peak area (n = 3) of the internal standard of peptide ITWDPPSSPVK and of the internal standard of peptide ASAHAITGPPTELITSEVTAR, respectively. Although the average peak area of the internal standard for peptide ASAHAITGPPTELITSEVTAR was equivalent in all subjects, the average peak area of the internal standard for peptide ITWDPPSSPVK was 8% in subject 4 and 1% in subject 7, and these values were less than 10% of those in other subjects, as shown in **Fig 2**.

**Fig 3:**
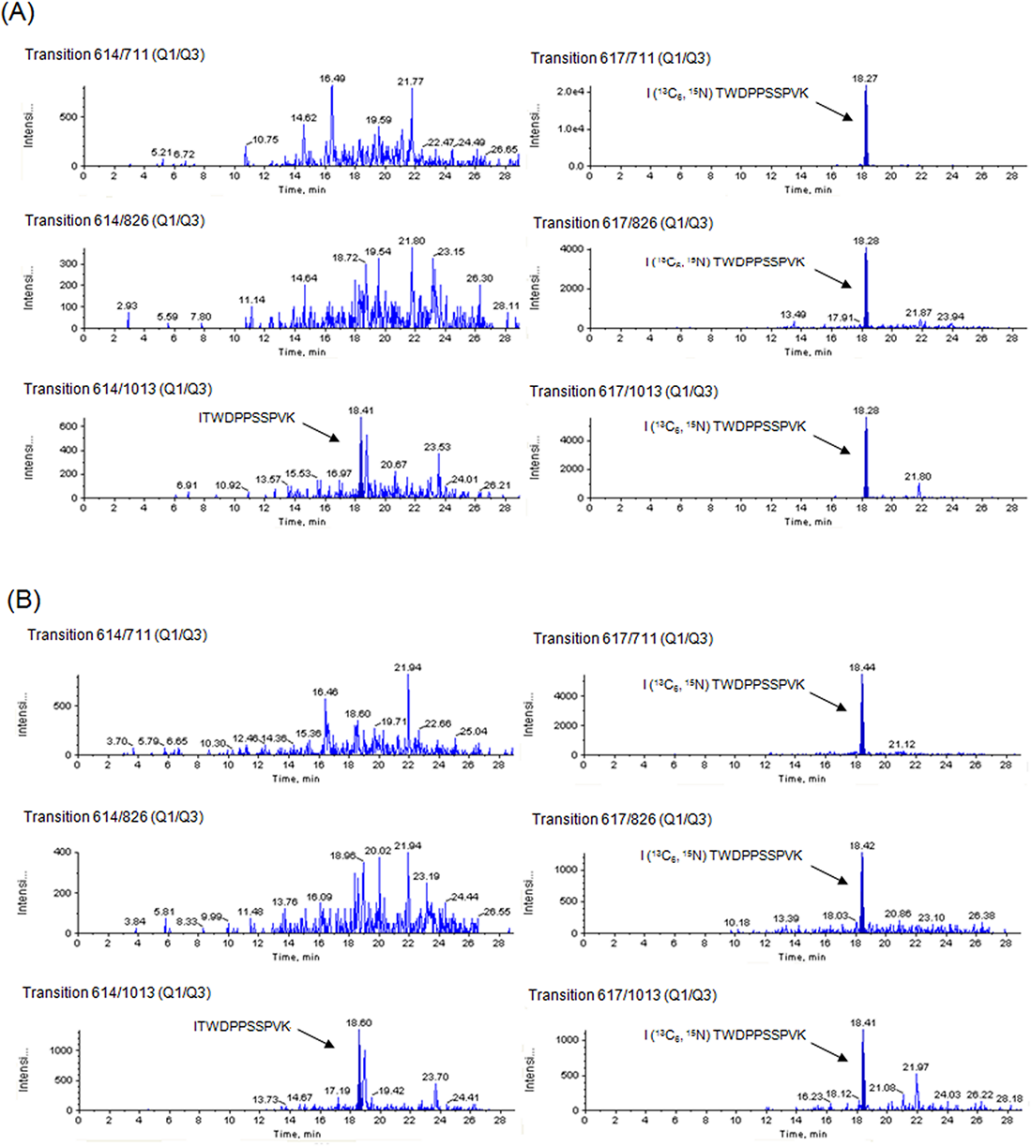
MRM chromatograms for peptide ITWDPPSSPVK of subject 4and subject 7. Transitions 614/711, 614/826 and 614/1013 were for endogenous peptide ITWDPPSSPVK, and transitions 617/711, 617/826, and 617/1013 were for stable isotope-labeled peptide, peptide I (^13^C_6_, ^15^N) TWDPPSSPVK. (A) subject 4 (B) subject 7. Only the endogenous peptide area of transition 614/1013 in subjects 4 and 7 was as high as that in other subjects, while other transitions (614/711 or 614/826) showed no peaks.

**Fig 3A and 3B** show representative MRM chromatograms for peptide ITWDPPSSPVK and the internal standard for subjects 4 and 7, respectively. As shown in **Fig 3**, only the endogenous peptide area of transition 614/1013 in subjects 4 and 7 was detected, while other transitions (614/711 or 614/826) showed no peaks.

Therefore, interfering substances with fragment ions of 614/1013 seemed to be eluted in peaks overlapping with peptide ITWDPPSSPVK, resulting in miscalculated erroneously high concentrations of the target peptide in subjects 4 and 7 due to the influence of interfering substances. Furthermore, the CVs of intra-assay reproducibility (n = 3) for transition 614/1013 in subjects 4 and 7 were 96.7% and 50.3%, which were extremely high (**Table 1**). These results indicate that transition 614/1013 has low specificity. By excluding the transition 614/1013, the concentrations of peptide ITWDPPSSPVK corresponded to below the detection limit in all measurements for subject 4 (**Table 1**). Furthermore the T-test *p* value for peptide ITWDPPSSPVK was changed from *p*< 0.05 to *p*<0.01, which correlated with peptide ASAHAITGPPTELITSEVTAR, as shown in **Fig 1A, 1A' and 1B**.

With respect to the intra-assay reproducibility (n = 3) of each transition, there were some subjects that were occasionally detected and occasionally not detected based on difference of the transition, because the detected values were near the detection limit. By the existence of samples not detected occasionally the average concentration of transitions was shifted to the lower, the difference between 2 transitions for peptide ITWDPPSSPVK was high (71.9% and 100.0%**)**, and the CVs among transitions for peptide ASAHAITGPPTELITSEVTAR were high (39.8–173.2%), as shown in **Table 1**.

By excluding the low-specificity transition 614/1013 and samples that were not detected in some measurements, the CVs of intra-assay reproducibility (n = 3) improved from 0.5–173.2 to 0.5–27.9 for peptide ITWDPPSSPVK, and from 2.7–173.2 to 2.7–34.6 for peptide ASAHAITGPPTELITSEVTAR (**Table 1**). Therefore, concentrations of transition 614/711 were used as the concentrations of peptide ITWDPPSSPVK and concentrations of transition 708/763 were used as the concentrations of peptide ASAHAITGPPTELITSEVTAR, since the transitions have high specificity and high sensitivity. That is, the concentrations of the best transition with high specificity and high sensitivity were used as the peptide concentrations, avoiding calculation of average from all transitions.

**Fig 4:**
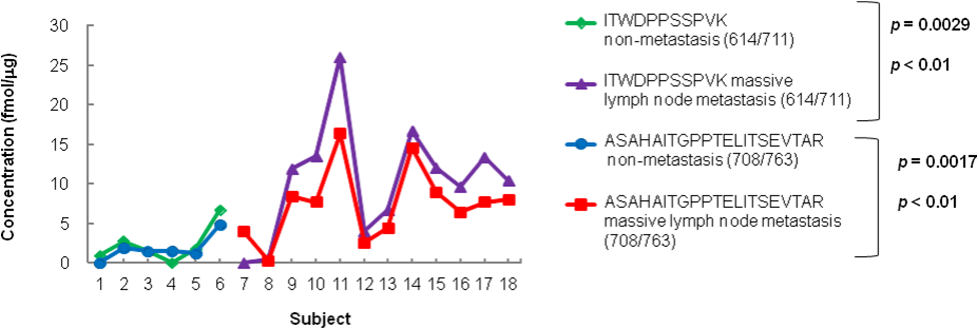
Type XIV collagen concentrations calculated from the best transitions of peptide ITWDPPSSPVK and peptide ASAHAITGPPTELITSEVTAR. After evaluation of imprecise transitions and using the best transition for the peptide concentration, concentrations of transition 614/711 were used as the concentrations of peptide ITWDPPSSPVK, and concentrations of transition 708/763 were used as the concentrations of peptide ASAHAITGPPTELITSEVTAR.

**Fig 5:**
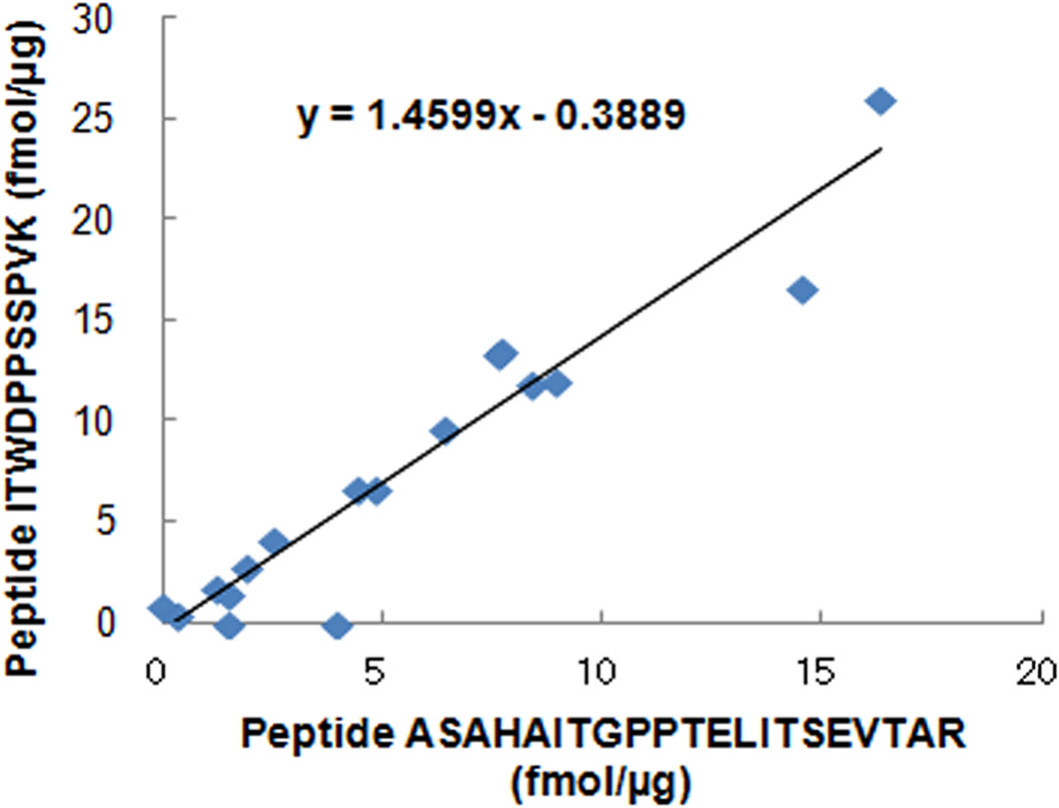
Correlation of type XIV collagen concentrations calculated from the best transitions of peptide ITWDPPSSPVK and peptide ASAHAITGPPTELITSEVTAR. The x-axis represents protein concentration of type XIV collagen calculated from the best transition (708/763) of peptide ASAHAITGPPTELITSEVTAR as determined by LC-MS/MS. The y-axis represents protein concentration of type XIV collagen calculated from the best transition (614/711) of peptide ITWDPPSSPVK as determined by LC-MS/MS. In the scatter plot, each data point represents protein concentration.

**Fig 4** shows the individual concentrations of peptide ITWDPPSSPVK and peptide ASAHAITGPPTELITSEVTAR after evaluation of imprecise transitions and using the best transition for the peptide concentration. The T test p-value for peptide ITWDPPSSPVK was changed from *p*<0.05 to *p*<0.01, which is equivalent to that for peptide ASAHAITGPPTELITSEVTAR. **Fig 5** shows that the individual concentrations calculated from the best transitions were correlated between peptide ITWDPPSSPVK and peptide ASAHAITGPPTELITSEVTAR (*p*<0.001; *r* = 0.9558). The results show that both peptides ASAHAITGPPTELITSEVTAR and ITWDPPSSPVK of type XIV collagen are specific to the massive lymph node metastasis group.

### 2. Validation by quantitative RT-PCR

**Fig 6:**
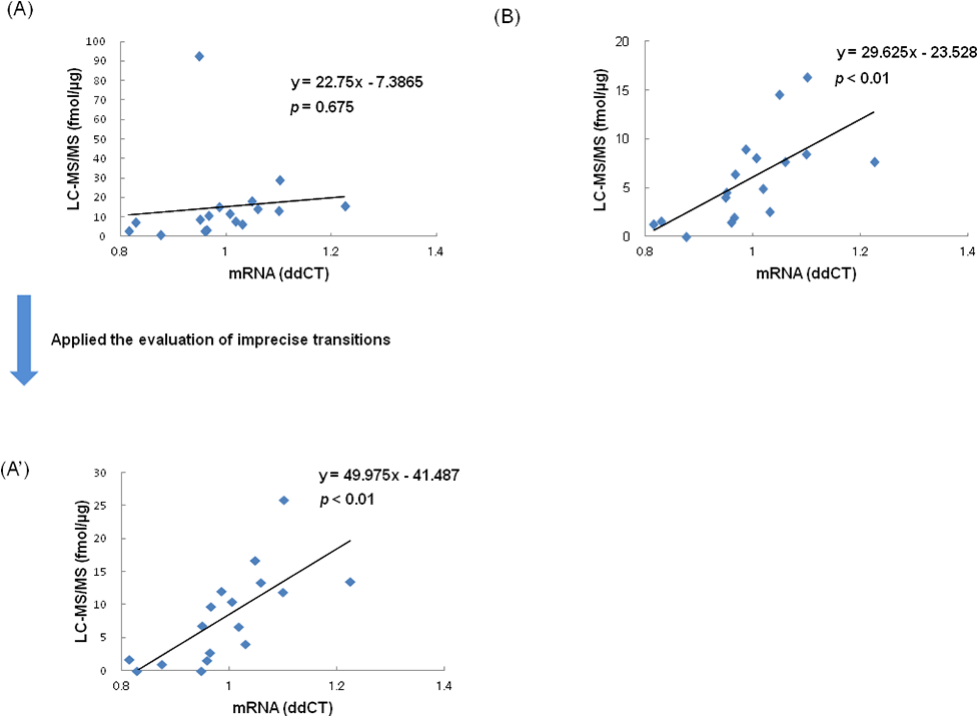
Correlation of concentrations between LC-MS/MS and quantitative real-time RT-PCR for validation of type XIV collagen. The x-axis represents m-RNA expression of type XIV collagen as determined by quantitative real-time RT-PCR. The y-axis represents protein concentration of type XIV collagen as determined by LC-MS/MS. In the scatter plot, each data point represents protein concentration and m-RNA expression. (A) The protein concentrations are average concentrations of all peptide transitions (614/711, 614/826, and 614/1013) of peptide ITWDPPSSPVK. (A') The protein concentrations are calculated from the best transition (614/711) for peptide ITWDPPSSPVK. (B) The protein concentrations are calculated from the best transition (708/763) for peptide ASAHAITGPPTELITSEVTAR.

The m-RNA expression of type XIV collagen was analyzed by quantitative real-time RT-PCR. **Fig 6** shows the correlation between type XIV collagen m-RNA expression and protein expression. One sample (Subject 8) was not analyzed by quantitative real-time RT-PCR due to insufficient quantity. Although protein expression calculated from the average concentration of all transitions including imprecise transition was not correlated with m-RNA expression (*p* = 0.675; *r* = 0.1099, Fig 6A), m-RNA expression was correlated with protein expression calculated from the best transition (614/711) for peptide ITWDPPSSPVK (*p*<0.01; *r* = 0.7190, Fig 6A') and with protein expression calculated from the best transition (708/763) for peptide ASAHAITGPPTELITSEVTAR (*p*<0.01; *r* = 0.6538, Fig 6B). The results indicate that type XIV collagen protein and m-RNA expression are correlated, and specific expression of type XIV collagen in massive lymph node metastasis was validated by quantitative real-time RT-PCR.

### 3. Identification of specific proteins

To examine the peptide-sequence specificity of type XIV collagen, a homology search was carried out for peptides ITWDPPSSPVK and ASAHAITGPPTELITSEVTAR by NCBI BLAST search against the human database. Only undulin and type XIV collagen share 100% amino acid sequence homology. Undulin is an alternative name for type XIV collagen, and the concentration of peptides ITWDPPSSPVK and ASAHAITGPPTELITSEVTAR represent the concentration of type XIV collagen.

## Discussion

In this study, type XIV collagen was identified as a protein specific for massive lymph node metastasis of breast cancer tissue as determined by quantitative LC-MS/MS and quantitative RT-PCR.

Type XIV collagen plays an adhesive role by integrating collagen bundles. It has been suggested that the large globular domain of Type XIV collagen protrudes from the bundles into the extracellular matrix where it interacts with cancer cells [26–28]. Although type XIV collagen may not be an appropriate target of antibody drugs because it is present in normal extracellular matrix, it may be a potential biomarker of metastasis and useful in evaluation of patient prognosis. Type XIV collagen has been previously reported to be expressed in odontogenic, brain, and pancreatic tumors [29], but not in breast cancer, and the significance of this expression remains unclear. Our findings suggest that type XIV collagen is a novel protein that is specific for massive lymph node metastasis breast cancer, in which it may play a role as an adhesion factor. Further studies on type XIV collagen are desired to verify its role as a prognostic factor and diagnostic marker for metastasis.

The 5 proteins aside from type XIV collagen that were expressed in massive lymph node metastasis of breast cancer as determined by MRM analysis without an internal standard included 2 types of type I collagen, MSTP161, angiomotin and hexokinase type I. Like type XIV collagen, type I collagen and MSTP161 are extracellular matrix proteins. The adhesive role of type XIV collagen is probably associated with the surface of collagen fibrils via type I collagen, and it might interact with other matrix molecules or cell surface receptors [30]. MSTP161 is also known as proline/arginine-rich end leucine-rich repeat protein (PRELP) or prolargin. PRELP binds collagen type I and type II through its leucine-rich repeat domain, and its function as a molecule anchoring basement membranes to the underlying connective tissue has been proposed [31, 32]. Angiomotin, localized on the cell surface, maintains tight junctions of protein complexes and regulates endothelial cell migration and tube formation [33, 34]. Therefore, the adhesive role of type XIV collagen may interact with type I collagen, PRELP and angiomotin. Hexokinase I is related to glycolysis [35]. Hexokinase II, which belong to the same hexokinase family, relates to invasion and metastasis because it facilitates and promotes the high-glycolytic tumor phenotype [36]. Further studies using LC-MS/MS quantification with internal standard and immunohistochemistry are desired to determine the specificity of these proteins in massive lymph node metastasis.

In the quantitative LC-MS/MS analyses, although type XIV collagen expression was as low as 5 fmol/μg even in tissue samples from patients with massive lymph node metastatic disease, this protein was quantified with high reproducibility by evaluation of imprecise transitions and using the best transition for the peptide concentration. The concentration distribution in individual subjects was correlated between 2 different peptides from the same protein. The results of the identification of type XIV collagen by quantitative LC-MS/MS correlated with the results of both quantitative real-time RT-PCR and immunohistochemistry. These finding also indicate the reliability and reproducibility of the quantitative LC-MS/MS method.

The results of peptide ITWDPPSSPVK analysis by LC-MS/MS in Subjects 4 and 7 showed that the influence of proteomic interference is a much greater challenge than that of small molecule quantification in samples that can be purified by deproteinization. Interference occurred at random in individual clinical samples and occasionally caused the miscalculation of erroneously high concentrations. When the presence of interference in peptides and subjects was not predicted in protein quantification, monitoring multiple transitions, evaluating imprecise transition using CV, and using the best transition for the peptide concentration were effective for high reliability quantification. Although 3 transitions per peptide were used in this study, it is feasible to use 5 transitions per peptide given the performance of the QTRAP5500, and this method can be used when a transition has reliable specificity. Our results indicate that when the transition has not been evaluated and its specificity is unknown, evaluation of imprecise transitions is required. Transition evaluation and peptide concentration calculation are common important points for protein quantification by LC-MS/MS in biological fluid.

## Conclusions

We developed quantitative methods for the analysis of type XIV collagen, and demonstrated that type XIV collagen is specific to massive lymph node metastatic breast cancer. In a future study, we hope to quantify type XIV collagen and specific candidate proteins in plasma and lymph fluid of both non-metastasis and massive lymph node metastasis tissues, with the ultimate goal being to utilize this technique to facilitate diagnosis and early detection, and to further our understanding of the mechanisms involved in metastasis. The correlation between the expression of specific proteins and their corresponding genes may contribute to the development of new drugs for the treatment of massive lymph node metastatic breast cancer. The protein was quantified with high reproducibility by evaluation of imprecise transitions and by using the best transition for peptide concentration. Analysis of imprecise transitions may also be useful for determining the pharmacokinetics of protein pharmaceuticals and the identification of protein biomarkers, as well as for the identification of specific proteins expressed in massive lymph node metastatic breast cancer.
